# One clone to rule them all: Culture-independent genomics of *Chlamydia psittaci* from equine and avian hosts in Australia

**DOI:** 10.1099/mgen.0.000888

**Published:** 2022-10-21

**Authors:** Rhys T. White, Susan I. Anstey, Vasilli Kasimov, Cheryl Jenkins, Joanne Devlin, Charles El-Hage, Yvonne Pannekoek, Alistair R. Legione, Martina Jelocnik

**Affiliations:** ^1^​ University of the Sunshine Coast, Centre for Bioinnovation, Sippy Downs, Sunshine Coast, Queensland 4557, Australia; ^2^​ The University of Queensland, School of Chemistry and Molecular Biosciences, Australian Infectious Disease Research Centre, Brisbane, Queensland 4072, Australia; ^3^​ The University of Queensland, Australian Centre for Ecogenomics, Brisbane, Queensland 4072, Australia; ^4^​ NSW Department of Primary Industries, Elizabeth Macarthur Agricultural Institute, Menangle, New South Wales 2568, Australia; ^5^​ The University of Melbourne, Melbourne Veterinary School, Asia Pacific Centre for Animal Health, Parkville, Victoria 3010, Australia; ^6^​ University of Amsterdam, Amsterdam UMC, Department of Medical Microbiology and Infection Prevention, Amsterdam 1105, The Netherlands

**Keywords:** *Chlamydia psittaci*, culture-independent sequencing, genomics, multi-locus sequence typing, phylogenetics, sequence type (ST)24

## Abstract

*
Chlamydia psittaci* is an avian pathogen with zoonotic potential. In Australia, *C. psittaci* has been well reported as a cause of reproductive loss in mares which subsequently have been the source of infection and illness in some in-contact humans. To date, molecular typing studies describe the predominant and clonal *C. psittaci* sequence type (ST)24 strains in horse, psittacine, and human infections. We sought to assess the clonality between ST24 strains and the emergence of equine ST24 with a comprehensive genomics approach. We used culture-independent probe-based and metagenomic whole-genome sequencing to investigate 13 *C *. *psittaci* genomes from horses, psittacines, and a pigeon from Australia. Published genomes of 36 *C *. *psittaci* strains were also used to contextualise our Australian dataset and investigate lineage diversity. We utilised a single-nucleotide polymorphism (SNP) based clustering and multi-locus sequence typing (MLST) approach. *C. psittaci* has four major phylogenetic groups (PG1-4) based on core-genome SNP-based phylogeny. PG1 contained clonal global and Australian equine, psittacine, and human ST24 genomes, with a median pairwise SNP distance of 68 SNPs. PG2, PG3, and PG4 had greater genomic diversity, including diverse STs collected from birds, livestock, human, and horse hosts from Europe and North America and a racing pigeon from Australia. We show that the clustering of *C. psittaci* by MLST was congruent with SNP-based phylogeny. The monophyletic ST24 clade has four major sub-lineages. The genomes of 17 Australian human, equine, and psittacine strains collected between 2008 and 2021 formed the predominant ST24 sub-lineage 1 (emerged circa 1979). Despite a temporal distribution of 13 years, the genomes within sub-lineage 1 had a median pairwise SNP distance of 32 SNPs, suggesting a recent population expansion or potential cross-host transmission. However, two *C. psittaci* genomes collected in 2015 from Victorian parrots clustered into distinct ST24 sub-lineage 4 (emerged circa 1965) with ovine strain C19/98 from Germany. This work describes a comprehensive phylogenomic characterisation of ST24 and identifies a timeline of potential bird-to-equine spillover events.

## Data Summary

The study sequences are available in the National Centre for Biotechnology Information (NCBI) under BioProject accession number PRJNA798154. Raw Illumina sequence read data generated in this study have been deposited to the NCBI sequence read archive (SRA (https://www.ncbi.nlm.nih.gov/sra)) under the accession numbers SRR17649252 to SRR17649264. A complete list of SRA accession numbers is available in Supplementary Materials, Table S1, (available in the online version of this article). In addition, the high-quality draft assemblies have been deposited to GenBank under the accession numbers JAKGCA000000000 to JAKGCM000000000. The versions described in this paper are versions JAKGCA010000000 to JAKGCM010000000. The programmes used to analyse raw sequence reads for polymorphism discovery and whole-genome sequencing-based phylogenetic reconstruction are available as described in the Methods. The authors confirm that all supporting data, code, and protocols have been provided in the article or supplementary data files. Supplementary Material for this article can be found on Figshare at 10.6084/m9.figshare.20348031 [1].

**Impact Statement**

*
Chlamydia psittaci* is a global avian pathogen with zoonotic potential. In Australia, *C. psittaci* has been well reported as a cause of reproductive loss in mares which subsequently have been the source of infection and illness in some in-contact humans. Genotyping and limited whole-genome sequencing analyses have elucidated the clonal nature of Australian *C. psittaci* sequence type (ST)24 equine, psittacine and human strains. Known challenges in culturing and isolation of chlamydiae, and sampling bias have hindered efforts to produce more *C. psittaci* genomes from Australia. However, culture-independent sequence enrichment of DNA extracted from diagnostic swab samples is becoming a popular alternative to conventional propagation in cell-based cultures. Our study expands the global *C. psittaci* genomes catalogue by providing whole-genome sequencing data of 13 horse, psittacine and pigeon strains from Australia over 6 years. Furthermore, we resolved the temporal and spatial relationship of the global collection of ST24, with collection dates spanning 1930 to 2021. We show that while contemporary ST24 are genetically distinct from other *C. psittaci* STs, ST24 has low pairwise SNP distances and likely diverged from a progenitor strain associated with the severe global outbreaks of human psittacosis in the 1930s.

## Introduction

*Chlamydia psittaci* is an avian pathogen thriving on the wildlife–livestock–human interface, and has been shown to infect over 450 species of birds, humans and, to a lesser extent, livestock (cattle, sheep, horses, and pigs) [2]. Molecular epidemiology has demonstrated an association of *C. psittaci* with the annual occurrence of late-term reproductive loss in mares in Australia [3–8]. In Australia, this intracellular pathogen is classified as a Risk Group 3, requiring the second-highest physical containment level (Physical Containment Level 3) [9]. Therefore, the isolation and culture of *C. psittaci* is not possible in many laboratories. Where isolation and culture are possible, it is laborious, costly, and often dependent on high infectious loads in the sample [10]. As such, culture-independent genomic and molecular typing research is called upon to answer several questions surrounding *C. psittaci* in the equine host.

Whole-genome sequencing (WGS) has been effective in global *C. psittaci* genomic studies using a larger collection of *C. psittaci* strains from a variety of livestock (sheep, cattle, pig), avian (psittacine, poultry, pigeons) and human hosts to discern phylogenetic relations between the strains and genome content [11–13]. Currently, there is limited *C. psittaci* genomic information from Australia, with only 12 draft genomes available from six human, one psittacine, and five equine *C. psittaci* strains [7, 14]. Comparative genomic and phylogenetic analyses revealed that all strains belong to the clonal ST24 clade, indicating that Australian *C. psittaci* strains are closely related, regardless of their hosts and/or location [7, 14]. Genomic studies have predicted that the global *C. psittaci* ST24 clade emerged from a common ancestor in the 1920s [13, 14]. However, the emergence of the equine ST24 strains in Australia has not been assessed concerning a time signal.

To date, extensive molecular typing using multi-locus sequence typing (MLST) and/or the major outer membrane protein (*omp*A) gene of Australian *C. psittaci* strains have provided strong evidence to support the hypothesised spillover events between the psittacine reservoir hosts and horses and/or humans in Australia, describing the dominant clonal ST24/*omp*A genotype A strains [4, 6–8, 15]. Furthermore, Australian studies also note a limited host range (mainly psittacine, equine, and humans) and limited sequence diversity, contrasting with the generalist host range and increased genetic diversity described globally [15–17]. Whilst the fine-detailed descriptions of genomic content or comprehensive phylogenies from WGS are not achievable with molecular typing, MLST phylogenetic analyses have proven highly congruent with WGS-based phylogeny [3, 7, 15], with MLST successfully distinguishing strains belonging to specific phylogenetic lineages [4, 8, 15]. Therefore, as the need for new and/or ongoing field studies to monitor the spread of *C. psittaci* in horses, livestock and/or wildlife increases, the utility of MLST for *C. psittaci* typing provides a highly reproducible and technically more accessible alternative to WGS.

This study sought to assess the clonality between global and Australian ST24 strains and their emergence with a comprehensive genomics approach. We used the culture-independent, probe-based sequence enrichment and Illumina HiSeq WGS to resolve new draft genomes of *C. psittaci* detected in swab samples from five psittacine, seven horse, and one pigeon host. Lastly, we assessed the congruency of *C. psittaci* MLST with WGS as a robust epidemiological tool for characterising infecting strains. This work will enable future genomics research that will aid in our understanding of the global expansion of ST24 and provide practical knowledge for *C. psittaci* epidemiology.

## Methods

### Sample descriptions used for whole-genome sequencing

Successful WGS (by Illumina HiSeq or probe capture enrichment followed by MiSeq) was achieved for a total of 13 previously described *C. psittaci-*positive swab samples collected from seven horse hosts, five psittacine hosts, and a pigeon host, across Australia in 2015 (*n*=2); 2018 (*n*=4); 2019 (*n*=2); 2020 (*n*=3); and 2021 (*n*=2) [15] (Supplementary Materials, Table S1) Successful WGS (using both host-depleted and probe-capture enrichment methodologies) was defined as spanning almost the whole chromosome with an average depth of ≥20-fold. These swab samples were processed by adding 300 µl of sterile TE (Tris-EDTA) buffer, vortexing, and heat lysis at 95 °C for 10 min, followed by DNA extraction using the QIAmp DNA mini kit (Qiagen, Australia), as previously described [15]. Before WGS, all DNA samples were once again screened with *C. psittaci* qPCR to estimate genome copy number [15, 18] (Supplementary Materials, Table S1), followed by DNA concentration and purity analyses using Qubit 3 Fluorometer and NanoDrop 1000 Spectrophotometer (both by ThermoFisher Scientific, Australia), respectively.

### Illumina HiSeq 2500 DNA sequencing

For initial WGS, we utilised seven previously described *C. psittaci* positive samples, including two samples (Qld_H_Foetus and Qld_H_Pl) from one horse host, two samples (Nsw_Dove_Liver and Nsw_Dove_Spleen) from one pigeon host, and three samples (401, 222L and 217) from three psittacine hosts [15] . The host methylated DNA was depleted by treating the DNA extracts from seven *C. psittaci* positive samples with the NEBNext Microbiome DNA Enrichment Kit (New England Biolabs, Ipswich, MA, USA), according to the manufacturer’s instructions. Following host DNA depletion, non-methylated DNA was subjected to multiple displacement amplification (MDA), using the REPLI-g kit (Qiagen, Australia) to increase bacterial DNA yield. The seven samples were sequenced as 2×150 bp paired-end reads on the Illumina HiSeq 2500 platform (Illumina Inc., San Diego, CA, USA) at the Australian Genome Research Facility (AGRF) (Parkville, Victoria, Australia). Only two samples from psittacine hosts (strains 401 and 222L) passed quality control (had ≥20-fold sequence coverage of a reference *C. psittaci* sequence) and were used for downstream analyses (Supplementary Materials, Table S1).

### *C. psittaci *-specific probe-based capture (SureSelect Targeted Enrichment)

For subsequent WGS using probe capture, 120-mer biotinylated RNA probes were designed with the assistance of Agilent Technologies (Agilent Technologies, Mulgrave, Victoria, Australia) using Tier2 design (0.5Mbp-2.999 Mbp) utilising two *C. psittaci* reference genomes (CP3; GenBank: CP003797 and Horse_pl; GenBank: CP025423) and the *C. psittaci* Horse_pl plasmid pCpHorse_placenta (GenBank: CP025424) to span the entire *C. psittaci* chromosome and plasmid. The probe sequences from this study are provided in Supplementary Materials (Tables S2 and S3. The custom-designed *C. psittaci* bait library was then synthesised by Agilent with SureSelect Capture Custom Probes - Tier two design parameters (0.5 Mbp – 2.9 Mbp capture size). The AGRF generated libraries targeting *C. psittaci* from a total of 16 DNA samples originating from 10 horse, four psittacine and two pigeon hosts using Agilent SureSelect XT HS2 library preparation with Agilent SureSelect XT HS Enzymatic fragmentation kit as per manufacturer’s instructions. Subsequent bait capture (performed in a batch of up to 16-plex) was completed with the Agilent SureSelect Capture Custom Probe kit as per manufacturer’s instructions. Briefly, the biotinylated RNA probes and SureSelectXT reagents were added to the DNA library, hybridising with the targeted chlamydial DNA. Before genome sequencing, magnetic separation was performed utilising streptavidin-coated beads to separate the hybridised DNA from the remaining complex DNA mixture.

### Illumina MiSeq DNA sequencing

Finally, captured libraries were assessed using an Agilent TapeStation D1000 assay and qPCR. Libraries were normalised and pooled, with 16 libraries sequenced as 2×150 bp paired-end reads on the MiSeq platform with V2-300 chemistry (Illumina Inc.) at the AGRF. Out of the 16 samples enriched using probe capture methods, sequence data from 11 DNA samples from seven horse hosts, three psittacine hosts, and a pigeon host passed quality control (high *C. psittaci* loads and ≥20-fold sequence coverage) and were used for downstream analyses (Supplementary Materials, Table S1).

### Dataset curation

To supplement the total of 13 draft genomes (two using host depletion and MDA enrichment and 11 using probe capture enrichment) assembled in this study (Supplementary Materials, Table S1), 36 publicly available *C. psittaci* genomes (15 complete genomes, 21 draft genomes) were included in downstream analyses (Supplementary Materials, Table S4). Of the 21 publicly available draft genomes, 16 had publicly available sequence read data which was downloaded from the National Centre for Biotechnology Information (NCBI) sequence read archive (SRA) using the ‘prefetch’ and ‘fastq-dump’ tools within the SRA Toolkit v2.9.0-mac64 (https://github.com/ncbi/sra-tools, accessed on 28 November 2021).

### Quality control of sequence data

The FastQC package v0.11.9 (http://www.bioinformatics.babraham.ac.uk/projects/fastqc/, accessed on 21 January 2022) was used to assess the sequence read data quality metrics for both the publicly available genomes with sequence read data available (*n*=16) and the genomes sequenced in this study (*n*=13). The taxonomic profiling tool Kraken v2.0.7-beta [19] was used with default parameters on the paired-end reads to screen the sequencing data for contamination against the NCBI Reference Sequence (RefSeq) database [20] for archaea, bacteria, human, viruses, and the ‘UniVec core’ subset of the UniVec database (a database of vector, adaptor, linker, and primer sequences). The sequence read data quality metrics for all 29 genomes are summarised in the Supplementary Materials (Table S5).

Using methods previously described [21], *C. psittaci* sequences for this study were obtained by using the Burrows–Wheeler Aligner (BWA) v0.7.17 [22] to map paired-end reads to the plasmid pCps6BC (GenBank: CP002587) and then subsequently to the chromosomes of 6BC (GenBank: CP002586); Horse_pl (GenBank: CP025423), Mat116 (GenBank: CP002744); 01DC11 (GenBank: CP002805); 02DC15 (GenBank: CP002806); 08DC60 (GenBank: CP002807); 84/55 (GenBank: CP003790); GR9 (GenBank: CP003791); MN (GenBank: CP003792); VS225 (GenBank: CP003793); WS/RT/E30 (GenBank: CP003794); M56 (GenBank: CP003795); WC (GenBank: CP003796); CP3 (GenBank: CP003797); NJ1 (GenBank: CP003798); and Rostinovo-70 (GenBank: CP041038). For each strain, *C. psittaci* sequence data was compiled into a single FASTQ file for each of the forward and reverse reads. These were then submitted to the NCBI SRA under BioProject accession number PRJNA798154.

### *De novo* genome assembly and *in silico* multi-locus sequence typing (MLST)

Following the workflow described by White and colleagues [21], Trimmomatic v0.36 [23] was used in paired-end mode to filter the raw reads by removing low-quality bases and read-pairs together with Illumina adaptor sequences. The filtered paired-end reads were then *de novo* assembled using MGAP (https://github.com/dsarov/MGAP---Microbial-Genome-Assembler-Pipeline, accessed on 21 January 2022), using the chromosome of the *C. psittaci* type strain 6BC as a closely related reference for scaffolding. Notably, the previously sequenced strains 8882_placenta and 9945_foetus [7] could now be resolved into the single chromosome and plasmid sequence, which underwent genome circularisation and annotation as described previously [21]. Assembly metrics were assessed using QUAST v5.0.2 [24]. *In silico* MLST was done using MLST v2.19.0 (https://github.com/tseemann/mlst, accessed on 21 January 2022) with default settings to query the assemblies against the Chlamydiales database hosted on PubMLST [25].

### Variant detection

HarvestTools v1.2 [26] was used to estimate the number of single-nucleotide polymorphisms (SNPs) and infer phylogenetic relationships. First, the 49 genome assemblies underwent a core-genome alignment using Parsnp v1.2, using Horse_pl as a reference [7]. When using Horse_pl as a reference for calling SNPs, 34 906 SNPs were identified across the 1 040 051 bp core-genome alignment. The Gubbins algorithm v2.3.4 [27] (default settings, ‘raxml mode’ with the General Time Reversible (GTR) GAMMA correction) was used to assess SNP density and/or predicted recombination regions in the core-genome alignment. Notably, SNPs identified in highly dense SNP and/or predicted recombination regions were removed (i.e. the Plasticity Zone (*n*=204 SNPs), major polymorphic membrane (*pmp*) protein loci (*n*=3159 SNPs), and type III secretion system loci (*n*=1233 SNPs), Supplementary Materials, Table S6), similar to the previous studies [7, 11, 12, 14]. The recombination blocks (identified by Gubbins), aligned with the assembly-based ML phylogeny generated from the alignment of core-genome SNPs, were visualised in Phandango v1.3.0 [28]. The final alignment used to reconstruct the *C. psittaci* phylogeny consisted of 30 310 core-genome SNPs.

### Phylogenetic analysis

The 30 310 core-genome SNP alignment was run through jModelTest v2.1.10 [29, 30], which identified the GTR nucleotide substitution model as the best-fit evolutionary model. Maximum Likelihood (ML) phylogenetic trees were reconstructed using RAxML v8.2.12 [31] (GTR-GAMMA correction) thorough optimisation of 20 distinct, randomised Maximum Parsimony trees before adding 1000 bootstrap replicates. To further identify robust Phylogenetic Groups (PGs) within the *C. psittaci* phylogeny, we used the 30 310 core-genome SNP alignment or the 3 098 bp MLST alignment as input into rheirBAPS v1.0.1 [32] (an R [33] implementation of hierarchical Bayesian Analysis of Population Structure (BAPS) [34]) with one level of clustering, allowing up to 10 initial clusters.

### Bayesian temporal analyses of the *C. psittaci* ST24 lineage

An ML phylogenetic tree comprised exclusively of 31 ST24 strains was reconstructed from an alignment of 420 core-genome SNPs using the above methods. Then, using the root-to-tip genetic distance in TempEst v1.5.15 [35], a regression analysis was used to determine whether there was a sufficient temporal signal in the data representing the *C. psittaci* ST24 lineage. A time-calibrated phylogenetic tree was generated with BEAST2 v2.6.6 [36] to further the temporal analysis. Next, we sought to determine whether the strict clock or uncorrelated relaxed clock (with a log-normal or exponential distributed rates) model best fit our dataset. Using the ‘tip dates’ function, nine models representative of a strict clock model, relaxed log-normal clock model, and relaxed exponential clock model were set up. Various population models were compared to ensure the selection of the best-fit model. These included the Bayesian skyline, coalescent constant, and exponential growth population size change models for each of the three clock models. The Gamma Site Model Category Count was set to four and the GTR substitution model rates determined from jModelTest were included (i.e. rate AC=1.60, AG=3.85, AT=0.50, CG=0.50, CT=3.94, and GT=1.00). The initial clock rate was set to 1.72×10^−3^ substitutions/site/year (estimated from the root-to-tip regression analysis in TempEst) with a uniform distribution and an upper bound of 0.1. All other priors were left as default. All models were tested with the Nested Sampling (NS) Bayesian computation algorithm v1.1.0 within the BEAST2 package with a particle count of 32, sub-chain length of 5000, and Epsilon of 1.0×10^−12^. This analysis provided evidence in favour of the uncorrelated relaxed clock model.

Once the best-fitting tree model was determined, three independent Markov chain Monte Carlo (MCMC) generations were conducted for 100 million generations for each analysis. Trees were sampled every 1000 generations, resulting in triplicate samples of 100 000 trees for each model test. To assess statistics, all BEAST2 runs were imported into Tracer v1.7.1 (http://github.com/beast-dev/tracer/, accessed on 21 January 2022). LogCombiner v2.5.0 (BEAST two package) combined the replicated analyses for each model with a 10 % burn-in to assess convergence. Finally, TreeAnnotator v2.4.5 (BEAST two package) removed the 10 % burn-in and generated maximum clade credibility (MCC) trees for each run (established from 243 million trees), reporting median values with a posterior probability limit set at 0.5. The resulting phylogenetic trees were visualised using FigTree v1.4.4 (http://tree.bio.ed.ac.uk/software/figtree/, accessed on 21 January 2022).

### *C. psittaci omp*A and MLST genotyping

Throughout 2021, new chlamydial equine reproductive loss cases in New South Wales (NSW) and Northeast Victoria were reported, and samples were submitted to the NSW Animal and Plant Laboratory for *C. psittaci* testing. To determine the infecting strain in these cases, *C. psittaci *-specific MLST and full-length *omp*A genotyping [15, 37] were applied to nine *C. psittaci *-positive samples from eight reproductive loss cases. This included seven samples from six cases from NSW and paired samples (Moranding_placenta3 strain from Victoria and MareA_W21 strain from NSW) of the WGS samples Morandig_Foal3_lung and FoalA_W21 from this study. The sample MareA_W21 was also included in WGS but failed quality control. In addition, a pooled sample from a diseased pigeon from NSW (Pigeon_NSW21) was available for genotyping. Metadata for all samples included host, year, geographical location, and source, can be found in the Supplementary Materials, Table S7.

All ten samples were processed, and DNA was extracted using QIAamp DNA Mini Kit (Qiagen, Germany), followed by *C. psittaci *-specific qPCRs to estimate genome copy number [18]. The *C. psittaci* MLST and *omp*A genotyping were performed as previously described [15, 37]. Upon PCR and sequencing of the *omp*A gene, the newly generated *omp*A sequences were subjected to Nucleotide-Nucleotide blast (BLASTn) [38] analysis to determine the *omp*A genotype, followed by deposition in GenBank under accession numbers: ON244501-ON244512. Similarly, after PCR and sequencing of MLST fragments, the infecting *C. psittaci* STs from the samples were determined by comparison against the *Chlamydiales spp*. database on PubMLST [25]. The sequenced MLST fragments, along with previously described and publicly available Australian and global *C. psittaci* STs, were concatenated into a 3098 bp alignment, which underwent phylogenetic reconstruction using ML (described above). The MLST sequences generated in this study were deposited in the *Chlamydiales spp*. database on PubMLST. To assess phylogenetic congruence between the MLST and WGS phylogenies for the same strains, Phylo.io v1.9 [39] was used to compare the ML trees generated from concatenated MLST genes and the core-genome SNPs.

## Results

### Culture-independent WGS of clinical *Chlamydia psittaci* strains

We aimed to obtain *C. psittaci* genomes without laborious cell culturing to assemble a set of strains representative of current clinical disease and/or infection. This study applied culture-independent WGS using *C. psittaci* baits on 16 *C *. *psittaci* positive DNA samples extracted from clinical swabs from horse and avian hosts and metagenomic sequencing on seven *C. psittaci* samples from swabs or liver tissues also from horse and avian hosts. WGS using custom-designed *C. psittaci* baits resulted in 11/16 samples (68.8%) sequenced and draft genomes produced, whereas WGS using metagenomic sequencing resulted in only 2/7 samples (28.6%) being successfully sequenced. The difference in success or failure based on the method used was not statistically significant (Fisher exact test for proportion, *P*=0.1688).

The resulting 13 draft genome assemblies sequenced in this study had a median total chromosome length of 1 167 334 bp (Interquartile range (IQR): 1 166 741 to 1 169 558 bp; range: 1 165 047 to 1 172 976 bp), a median GC content of 39.04 % (IQR: 39.04–39.05 %; range: 39.00–39.06 %), and a median N50 statistic of 327 129 bp (IQR: 285 565 to 769,768 bp; range: 100 441 to 777 346 bp) (Supplementary Materials, Table S8). Notably, 12/13 (92.3 %) genomes were ST24/*omp*A genotype A, with only strain Racing_Pigeon24 characterised as ST27/*omp*A genotype B. *De novo* assembly of the chlamydial plasmid resulted in a single 7 553 bp contig for all, except Mare_F43_pooled strain (GenBank: JAKGCG000000000) where plasmid *de novo* assembly resulted in three contigs. For strain Plac_F43, the plasmid was 7 552 bp in length due to a 1 bp deletion (3070 del A) in the intergenic region upstream of the virulence plasmid protein pGP4.

Jenkins and colleagues [7] performed WGS and provided preliminary draft assemblies of the first equine *C. psittaci* genomes. In addition to the new WGS set from our study, this study also provides the closed genomes of the previously sequenced strains 8882_placenta (GenBank: CP092197) and 9945_foetus (GenBank: CP092199). *De novo* assembly successfully resulted in a single circular chromosome of 1 166 642 bp and 1 166 085 bp for 8882_placenta and 9945_foetus, respectively, with both strains carrying the 7 553 bp chlamydial plasmid (Supplementary Materials, Fig. S1). General features, such as the number of predicted coding DNA sequences (CDS) and GC content, are consistent across each strain and are listed in (Supplementary Materials, Table S9).

### Global *C. psittaci* phylogeny confirms the clonality within genetically diverse species and is congruent with MLST phylogeny

The genomic diversity of 13 Australian *C. psittaci* from this study was assessed by comparisons to 36 publicly available *C. psittaci* genomes. This data set includes major known *C. psittaci* genotypes collected over 90 years from a range of hosts and provides an opportunity to answer fundamental phylogenomic questions about this critical veterinary pathogen (Supplementary Materials, Table S8).

**Fig. 1.**
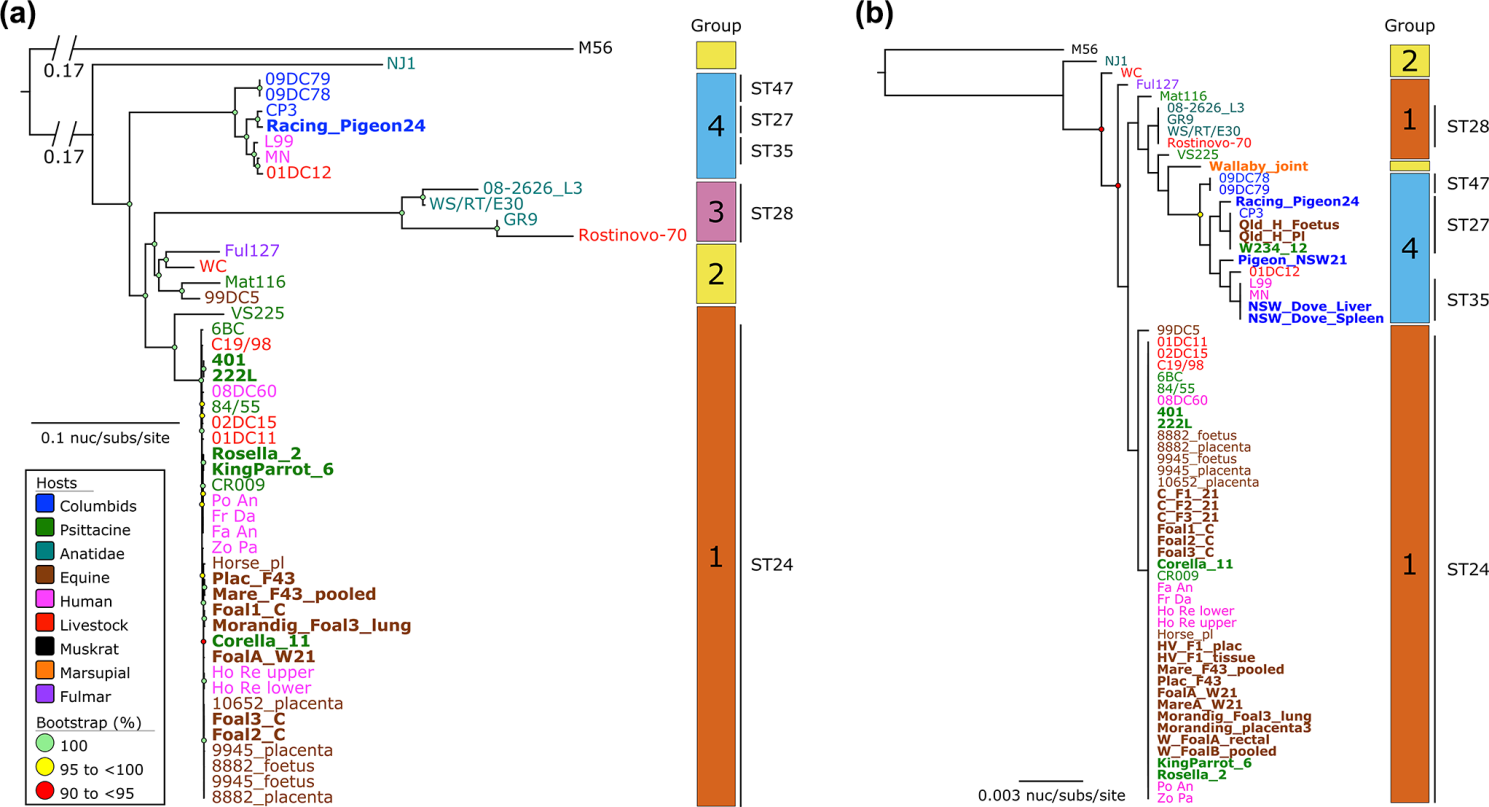
Phylogenetic relationships of *Chlamydia psittaci *. (a). Maximum likelihood phylogeny inferred from 30 310 core-genome single-nucleotide polymorphisms (SNPs) called from 49 genomes. The 30 310 SNPs were derived from a core-genome alignment of 1 040 051 bp and are called against the reference chromosome Horse_pl (GenBank: CP025423). (b). Maximum likelihood phylogenetic analysis of a 3 098 bp alignment representing concatenated MLST sequences. Both phylogenies are midpoint rooted. Branch lengths represent the nucleotide substitutions per site, as indicated by the scale bar. The branch bearing double hatch marks indicates that it has been truncated and is not proportional to the rest. Bootstrap values (using 1000 replicates) are shown. Strains from this study are in bold. The outer blocks reflect hierBAPS defined Phylogenetic Groups (PGs). Major ST is denoted next to the PGs.

We investigated the phylogenetic relationships and diversity of these 49 genomes using two approaches: a core-genome SNP phylogeny and a MLST phylogeny (Fig. 1). To assess SNP density and/or predicted recombination regions, we analysed the core-genome alignment using Gubbins. This predicted eight substantial regions of recombination, with high SNP densities in and surrounding the major *pmp* gene clusters, type III secretion system loci, *omp*A region, and the plasticity zone (Supplementary Materials, Fig. S2). An ML phylogenetic tree was inferred from an alignment of 30 310 core-genome SNPs (excluding regions of high genome plasticity) (Fig. 1a). To unambiguously identify robust PGs within the *C. psittaci* phylogeny, we used rheirBAPS with one level of hierarchical clustering to further define genetic clusters based on SNP and MLST-derived phylogenies. For the SNP-derived phylogeny, rheirBAPS identified four almost identical primary PGs. The strains VS225 and all ST24 formed PG1; diverse STs from fulmar, livestock, horse and including NJ1 and M56 formed PG2; ST28 formed PG3, and finally, pigeon-type STs formed PG4 (Fig. 1a). Based on our visual inspection, the core-genome SNP tree showed that these four distinct PGs (with 100 % bootstrap support) comprise of genetically diverse and clonal strains collected from different hosts, like previous studies [7, 12–14].

PG1, apart from the psittacine strain VS225 (ST204, *omp*A genotype F), contained a monophyletic clonal lineage consisting exclusively of ST24, *omp*A genotype A genomes (*n*=31) (Fig. 1a). All 25 Australian psittacine, human, and horse ST24 strains from this and previous studies cluster within this clonal lineage, in addition to five strains isolated in Europe and the type strain 6BC, collected in 1930 from North America. Notably, this clonal ST24 lineage had a median pairwise SNP distance of 68 SNPs (IQR: 39 to 79; range: 0 to 107 SNPs).

PG2 strains were more genetically diverse, resolving into at least two sub-clades (Fig. 1a). Sub-clade I contained strains from a juvenile *Fulmarus glacialis* (fulmar) (ST197) and cattle (ST32) that had a pairwise SNP distance of 1339 SNPs, and Sub-clade II contained strains from a horse from Germany (ST218) and psittacine from Japan (ST208) that had a pairwise SNP distance of 938 SNPs. Although clustered within PG2, M56 and NJ1 clustered into each own separate lineage.

However, PG3 were all ST28 strains from duck and livestock hosts with different *omp*A genotypes (Supplementary Materials, Table S7). The ST28 clade has greater genomic diversity than the other two clades, with a median pairwise SNP distance of 3158 SNPs (IQR: 1917 to 4088; range: 1289 to 5356 SNPs).

PG4 contained six strains from pigeon, human and livestock hosts, including the Australian Racing_Pigeon24 strain (Fig. 1a). These strains comprised multiple STs (ST47, ST27, and ST35) and *omp*A genotypes B and B/E (Supplementary Materials, Table S7), with the median pairwise SNP distance of 651 SNPs (IQR: 527 to 968; range: 2 to 1095 SNPs). The pigeon-associated strains Racing_Pigeon24 (Australia) and CP3 (United States) clustered in an ST27 sub-clade and are separated by only 193 SNPs.

We also employed the MLST-derived phylogeny to assess the clustering of the: (i) *C. psittaci* genomes analysed in this study; (ii) additional publicly available STs from a range of hosts; and (iii) the newly typed strains into different groups of phylogenetic relevance (Fig. 1b). For MLST-derived phylogeny, rheirBAPS identified three primary PGs, where 99DC5, all ST24, Ful127, Mat116, and all ST28 formed PG1, PG2 grouped all remaining genetically diverse STs from a wallaby, turkey, and livestock. In contrast, pigeon-type STs (ST 27, 35, and 47) formed PG4 (Fig. 1b). Not surprisingly, additional horse *C. psittaci* strains (including the paired samples (Moranding_placenta3 from Victoria and MareA_W21 from NSW) of the WGS samples Morandig_Foal3_lung and FoalA_W21) were all ST24, and *omp*A genotype A. Strain Pigeon_NSW21 was a novel ST320 and *omp*A genotype B and clustered with other genetically similar pigeon types (ST27, ST35, and ST47 and *omp*A genotypes B and B/E) strains (Fig. 1b).

To investigate the level of congruence between SNP-derived phylogeny and MLST phylogeny for the same strains, we used the Phylo.io web tool. Side-by-side tree comparison and degree of correspondence between the nodes using Jaccard scores showed that subtree structures of SNP-derived and MLST ST24 and pigeon-type clades were 100 % identical (with a score of 1). The rest of the diverse STs subtree structures achieved ~50 % similarities between the nodes (Supplementary Materials, Fig. S3). We evaluated the MLST approach through comparisons with the SNP-based inferences. We showed that, for the most part, and major PGs (ST24 and pigeon-type STs), the clustering of *C. psittaci* by MLST was congruent with core-genome SNP phylogenies (Figs 1 and S3).

### The evolutionary timeline of *C. psittaci* ST24 is congruent with previous studies

**Fig. 2.**
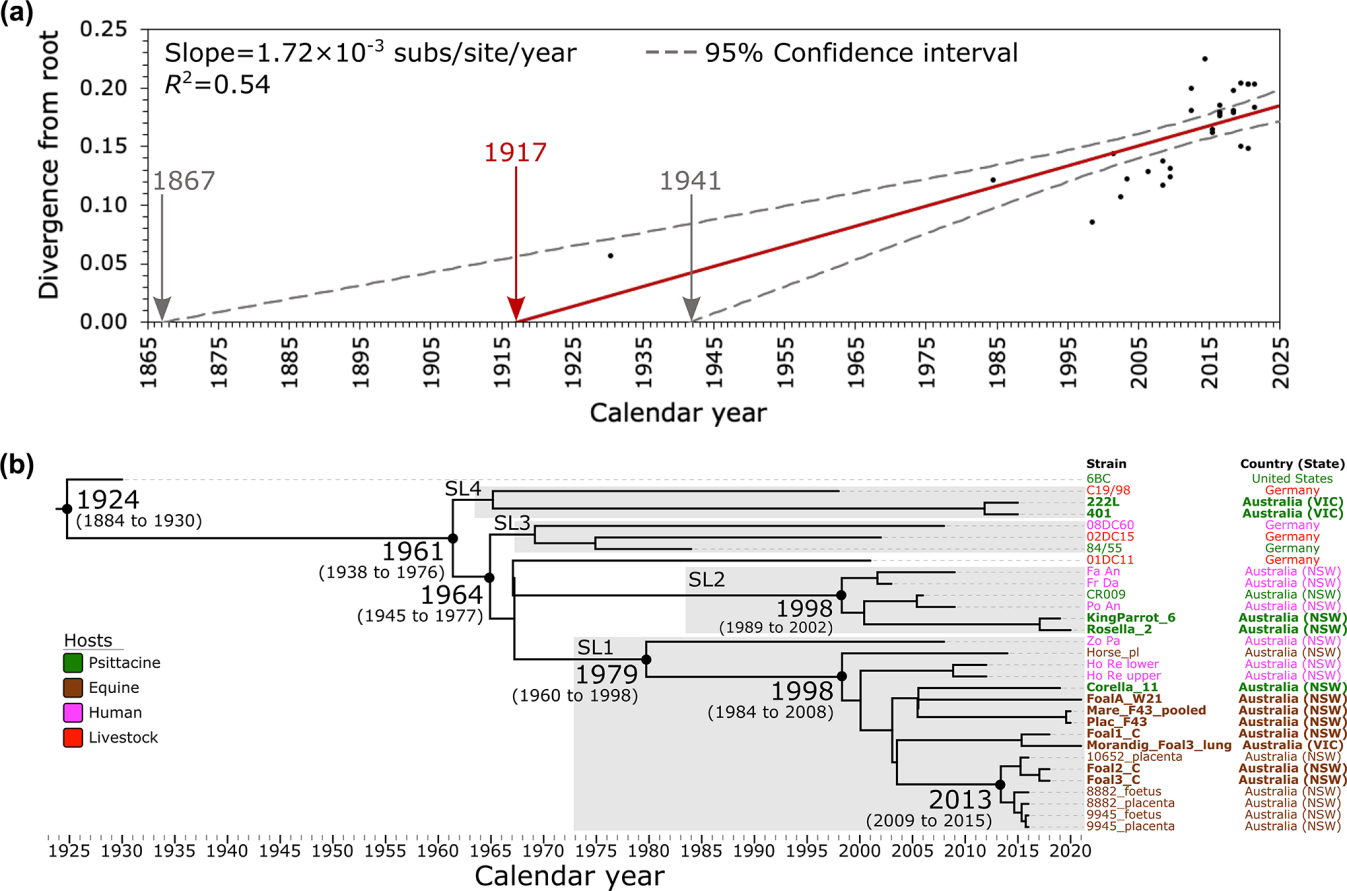
Evolutionary reconstruction of *Chlamydia psittaci* sequence type (ST)24. (a). Linear regression of root-to-tip genetic distance plotted against the collection year implemented in TempEST. The nucleotide substitution rate is indicated by the slope of the solid red regression line and is supported by 95 % confidence intervals (grey dashed lines). (b). A time-calibrated maximum clade credibility tree was inferred from 420 core-genome single-nucleotide polymorphisms (SNPs) called from 31 ST24 genomes. The 420 SNPs represent SNPs outside the Plasticity Zone, major polymorphic membrane protein regions, and type III secretion system. SNPs were derived from a core-genome alignment of 1 027 103 bp and are called against the reference chromosome Horse_pl (GenBank: CP025423). The X-axis represents the emergence time estimates. Strains from this study are in bold. Major ST24 sub-lineages (SL)1–4 are shown.

Given the low genetic diversity of the ST24 clade, we next sought to investigate the level of this clonality. Using ST24 strain Horse_pl as a reference, an ML phylogeny was inferred from an alignment of 420 core-genome SNPs (excluding regions of high genome plasticity) (Supplementary Materials, Fig. S4). The ST24-only SNP-based phylogeny was congruent with *C. psittaci* species-level phylogeny in that the median pairwise SNP distance between ST24 strains is 67 SNPs (IQR: 40 to 79; range: 0 to 104). To investigate the temporal signal of our dataset, the ML phylogeny was input into TempEST. The divergence time and evolutionary distance for the 31 ST24 genomes showed a linear relationship (correlation coefficient=0.74), with the regression analysis in TempEst indicating that the ST24 genomes in our dataset accumulate mutations at a rate of 1.72×10^−3^ substitutions per site per year (*R^2^* =0.54) (Fig. 2a). Although the approach with TempEst is an exploratory analysis, it suggests that this dataset exhibits a clock-like behaviour. Based on this root-to-tip divergence analysis, the most recent common ancestor (MRCA) to the ST24 lineage is estimated to have emerged in 1917 (95 % confidence interval: 1867 to 1941).

With an appropriate temporal signal detected in the ST24 dataset, we utilised the NS Bayesian computation algorithm to determine the best-fitting tree model to generate a time-measured phylogeny. These analyses supported the relaxed log-normal clock model with the Bayesian skyline population size change model based on a marginal likelihood estimate of −2 948.27 (standard deviation: ±2.03) (Supplementary Materials, Table S10). Like the root-to-tip divergence analysis, using median node heights, BEAST2 pinpoints the time to the MRCA of the ST24 lineage to 1924 (95 % highest posterior density (HPD): 1884 to 1930) (Fig. 2b). The median evolutionary rate determined by BEAST2 is 2.00×10^−3^ substitutions per site per year (95 % HPD: 1.21×10^−3^ to 2.76×10^−3^), which is also consistent with the root-to-tip divergence analysis. Our dataset describes one SNP for every 2445.5 bases across the 1 027 103 bp core-genome to correct for ascertainment bias. This translates to a genome-wide mutation rate of 8.19×10^−7^ mutations/year/site, relative to genome size, for the ST24 lineage, in concordance with previous studies [13, 14].

### Fine-detail resolution of the clonal ST24 clade: a lineage persisting in Australia since at least the 1960s

Phylogenetic inference in BEAST2 resolved fine-detail relationships among the ST24 strains, establishing four major sub-lineages (here termed ST24 clades sub-lineage (SL)1 to 4) (Fig. 2b). SL1 is the predominant sub-lineage which contains 17 Australian genomes, with a median pairwise SNP distance of 32 SNPs evenly distributed across the chromosome (IQR: 24 to 42; range: 0 to 63). Of these 17 genomes, 13 are from horse hosts, three from humans, and one from psittacine. Notably, the maximum pairwise distance of 63 SNPs is between the strain Zo Pa (human, isolated in 2008 from Blue Mountains region, NSW) and the first described horse reproductive loss strain Horse_Pl (Horse, isolated in 2014 from Wagga Wagga region, NSW). We estimate that the MRCA for this SL1 occurred circa 1979 (95 % HPD: circa 1960 to 1998). Relative to Horse_pl, using the pairwise SNP data, 27 SNPs define the terminal branch of the 16 genomes with a common ancestor that existed circa 1998 (95 % HPD: 1984 to 2008) (Supplementary Materials, Table S11).

Regarding the horse strains within SL1, we observe four distinct clusters. Strain Horse_Pl, detected in 2014 from Wagga Wagga, NSW, formed its distinct lineage, where the common ancestor emerged circa 1998 (95 % HPD: 1984 to 2008). The rest of the horse strains formed at least three sub-lineages from two geographically different regions in Australia, NSW and Victoria. The first horse sub-lineage, with predicted emergence circa 2005 (95 % HPD: 2003 to 2005) clustered FoalA_W21 strain collected 2021 from Wagga Wagga region, NSW and paired strains Mare_F43_pooled and Plac_F43 collected in 2020 from Hunter Valley region in NSW, but also strain Corella_11, collected from a psittacine host from far west NSW, in 2019 (Fig. 2b). This sub-lineage shows genetic diversity, with a pairwise distance of 34 SNPs between strain Corella_11 and FoalA_W21 and 42 SNPs between strain Corella_11 and both strains Mare_F43_pooled and Plac_F43. Not surprisingly, the paired strains Mare_F43_pooled and Plac_F43 are identical based on this ST24 core-genome analysis (0 SNPs).

The second horse sub-lineage, with predicted emergence in 2015 (95 % HPD: 2010 to 2017), clustered strains Foal1_C, collected in 2018 from the Hunter Valley in NSW, and the Morandig_Foal3_lung strain, collected in 2021 from Northeast Victoria. A pairwise distance of only eight SNPs between strain Foal1_C and Morandig_Foal3_lung indicates that this sub-lineage has likely persisted and has been detected in Victoria.

Finally, the most predominant third horse sub-lineage is represented by five strains (10652_placenta, paired 8882_foetus and 8882_placenta, and paired 9945_foetus and 9945_placenta) from 2016 and two strains from the same farm in 2018 (Foal2_C, Foal3_C), all from the sympatric region within Hunter Valley, NSW, where the common ancestor emerged circa 2013 (95 % HPD: 2009 to 2015) (Fig. 2b). This lineage has low genetic diversity, with a median pairwise distance of two SNPs (IQR: 1 to 3; range: 0 to 6). The maximum pairwise SNP distance (6 SNPs) is between strains 8882_foetus and Foal3_C. It is worth noting that Foal1_C was from the same farm in 2018 as the strains Foal2_C and Foal3_C. However, it clustered into the second sub-lineage with the Victorian horse strain from 2021.

SL2 contained six *C. psittaci* strains from three human and three psittacine hosts in Australia, from the same *C. psittaci* endemic Blue Mountains region, NSW, with a median pairwise distance of 13 SNPs (IQR: 8 to 16; range: 1 to 18). The psittacine CR009 and human Fr Da, Fa An and Po An strains were collected in 2003 and 2009, whereas psittacine strains KingParrot_6 and Rosella_2 were collected in 2019 and 2020, respectively. We estimate the divergence between SL2 and other ST24 sub-lineages from circa 1998 (95 % HPD: 1989 to 2002). SL3 contained three *C. psittaci* strains from livestock, human, and psittacine hosts from Germany. SL3 has higher genomic diversity when compared with SL1 and SL2, with a pairwise distance ranging between 25 and 45 SNPs. SL4 comprises three strains, notably clustering *C. psittaci* from a sheep C19/98 strain from Germany, collected in 1998, and two psittacines from the same region in Victoria, collected in 2015. The pairwise distances suggest lower genomic diversity between the two Australian strains (5 SNPs) compared with the sheep strain from Germany (maximum distance of 55 SNPs). Relative to Horse_pl, using the pairwise SNP data, 44 SNPs and one insertion define the terminal branch to strains 401 and 222L (Supplementary Materials, Table S12).

## Discussion

To date, and in the absence of infection studies, the enigmatic nature of *C. psittaci* ST24 strains associated with Australian equine reproductive loss was elucidated using genotyping and limited WGS analyses. Like Australian human zoonotic infections, these studies also support that the horse *C. psittaci* infections result from spillover (direct and/or indirect) from psittacines [3, 4, 6–8, 14, 15]. Our study expands the global catalogue of *C. psittaci* genomes by providing contemporary WGS data of seven equine, five psittacine, and one pigeon strain from Australia and resolves the emergence of these strains in an Australian setting.

Previous attempts to generate more Australian equine (but also avian and human) *C. psittaci* genomes have been hampered by chlamydial isolation and WGS technical challenges. Recent metagenomic sequencing of DNA extracted from equine foetal tissues and membranes without enrichment accurately detected *C. psittaci* only and at a high relative abundance; yet, it could not achieve suitable read numbers for any genomic level work [40]. Culture-free sequencing of DNA extracted from diagnostic swab samples is becoming increasingly popular in the chlamydial field. It allows for direct characterisation of the infecting strain and real-time understanding of epidemiology, diversity and/or evolution [14, 41–43]. In our study, custom-designed probe-based capture resulted in successful WGS of ~70 % (11/16) of our samples, compared to only ~30 % (2/7) using host methylated DNA depletion-microbial DNA enrichment WGS. Here, the limited success of the microbial enrichment WGS could have resulted from the multi-step sample preparation, technical problems during sequencing, or degraded nucleic acids [10]. While the probe-based capture method addressed the challenges above, it was more costly than the metagenomic WGS. A limitation of our study is that both WGS methods failed to capture genomes of the low load *C. psittaci* (10–50 copies/μl DNA) samples. In both cases, we lost several potentially epidemiologically significant strains, including an equine ‘pigeon-type’ ST27 strain from the isolated case of equine reproductive loss in 2017 in a northern region of Australia (Queensland), an ST35 strain from a native spotted dove from NSW from the early 2000s [4], additional psittacine ST24 strains from Victoria also collected in 2015, a paired sample (MareA_W21) to genome resolved FoalA_W21, and an ST24 strain detected in a healthy foal [5].

Despite our dataset mainly consisting of the clonal horse and psittacine *C. psittaci* strains, we revealed several interesting observations. Our phylogenetic analyses: (i) reiterate the clonality and generalist host nature of the ST24 lineage; (ii) support lower genetic diversity and host association within the ‘pigeon-type’ PG4, where our pigeon strain RacingPigeon_24 clustered; and (iii) demonstrate that overall, the *C. psittaci* population is genetically diverse in agreement with previous studies [7, 11, 13, 14, 17]. Whilst we have now expanded Australian psittacine *C. psittaci* ST24 genomes from one (CR009) to six, provided the first pigeon *C. psittaci* from Australia and confirmed that the ST24 clonality of the equine reproductive loss strains, another limitation of our study was that we were not able to obtain additional samples containing previously genotyped contemporary diverse strains from other Australian non-psittacine, human, livestock and/or marsupials hosts [15, 44, 45].

Comparing the MLST to the SNP phylogeny shows that *C. psittaci* MLST broadly reflects the core-genome phylogeny. Despite its limited phylogenetic resolution, MLST (particularly when supplemented with *omp*A genotyping) remains helpful in field studies to characterise infecting strains in real-time. In most settings, molecular detection (qPCR) surveillance should be complemented with epidemiological investigations with MLST to monitor the emergence of new strains that may cause epizootics in intense animal management systems, like the Thoroughbred breeding industry [5]. The threat to late Thoroughbred pregnant mares in the Hunter Valley (ST24 endemic), from a different ST with the same pathology, is real as the movements of Thoroughbred horses between states and/or countries is quite common. MLST-derived phylogeny would also be useful to avoid the need for core-genome phylogenetics for established and/or closely related STs (such as ST24 or genetically similar pigeon types) [46].

Unsurprisingly, comprehensive phylogeny clustered all equine and psittacine *C. psittaci* strains from this study into the clonal monophyletic ST24 lineage. Nevertheless, fine-detailed phylogenetic and temporal analyses of the ST24 revealed interesting observations on the structure of this predominant lineage. The evolutionary stable ST24 lineage was further divided into four distinct sub-lineages, where Australian strains dispersed over at least three sub-lineages. To some extent, we also observed that geographical location is associated with phylogenetic distance in ST24, as seen in SL4, consisting of two psittacine strains collected in 2015 from the same region in Victoria, SL2 consists of human and psittacine strains collected from 2003 to 2020 from the endemic *C. psittaci* Blue Mountains region, and SL1 cluster consisting of Hunter Valley equine strains collected in 2016 to 2020 from at least four farms within a 100 km radius. This geographical phylogenetic association may indicate a rapid expansion and/or contemporary spillover of ST24 sub-clones over a similar period, as observed in other chlamydial species [13, 14, 47].

All the equine *C. psittaci* strains clustered into SL1 with other Australian psittacine and human *C. psittaci* strains; but they also formed sub-lineages consisting of equine *C. psittaci* strains only. Our analyses predicted that the MRCA for this SL1 was circa 1979 (95 % HPD: circa 1960 to 1998). While any phylogenomic dating should be interpreted carefully, we can postulate that the lineage of these equine *C. psittaci* strains (and other human and psittacine from SL1) has existed in Australia since the 1960s (at least). A recent retrospective study has also provided evidence that this pathogen has been a cause of sporadic equine reproductive loss in Australia for at least >30 years since the early 1990s [8], highlighting the potential underreporting of equine *C. psittaci* infections. Furthermore, the global movement of horses for breeding, training and/or competition purposes is a common practice [48]. However, such practice may facilitate the unconstrained international transmission of chlamydial infections, such as observed for *Streptococcus equi* subspecies *equi *, the causative agent of strangles [49]. As such, the equine *C. psittaci* sub-lineages represent contemporary strains causing reproductive loss across Australia.

Despite the widely supported *C. psittaci* spillover from psittacine to horses as the origin of this infection in horses, in our study, we likely did not sample that specific lineage, as only one sub-lineage clustered directly psittacine strain Corella_11 and three equine *C. psittaci* strains. In Australia, horses originally consisting of English Thoroughbred and Spanish stock were introduced with the arrival of the British First Fleet in 1788 [50]. In contrast, Australia has long been known as the ‘Land of Parrots’, with >56 parrot species (including known *C. psittaci* hosts cockatoos, lorikeets, rosellas, and budgerigars) possibly originating and found across Australia [51]. Furthermore, *C. psittaci* infections in Australian parrots were highlighted in the early 1930s in work by Sir Frank Macfarlane Burnet and colleagues [52, 53], which coincided with severe global outbreaks of human psittacosis in Europe, North and South America [2, 54]. Considering the above, however, in the absence of genomic data from these historical strains, we can further speculate that spillover to both horses and humans in Australia could have occurred at a similar time as predicted in our study for the MRCA of the ST24 lineage in 1924 (95 % HPD: 1884 to 1930).

Molecular and/or cell biology/infection studies to dissect the significant pathogenicity of *C. psittaci* and to understand the risk posed by *C. psittaci* to livestock and humans are being increasingly conducted in Australia and worldwide [7, 11, 14, 15, 55–57]. In Australia, further studies must focus on understanding the pathogenicity of *C. psittaci* strains causing equine reproductive loss and the factors that increase the risk of infection spillover [5] but also to assess the genetic diversity of *C. psittaci* in the broader range of avian and non-avian hosts, including humans [14, 15, 44, 45, 58]. The opportunities for ongoing spillover due to habitat degradation/encroachment (or increase) of the wildlife/livestock interface and reduced food/water resources for birds driving them into paddocks to feed on horse food are plausible [59]. Therefore, the One Health approach must involve surveillance, infection studies and molecular research, and responses to chlamydiosis by public health and veterinary authorities [15, 45, 58] to continue the long-established Australian research into this critical globally disseminated zoonotic pathogen.

## Supplementary Data

Supplementary material 1

Supplementary material 2

Supplementary material 3

Supplementary material 4

Supplementary material 5

Supplementary material 6

Supplementary material 7

Supplementary material 8

Supplementary material 9

Supplementary material 10

Supplementary material 11

Supplementary material 12

Supplementary material 13
