# Supplementary material for: One clone to rule them all: Culture-independent genomics of Chlamydia psittaci from equine and avian hosts in Australia
Source: Microb Genom. 2022 Oct 21;8(10):mgen000888. doi: 10.1099/mgen.0.000888 (PMC9676050; doi:10.1099/mgen.0.000888)
Supplement: Supplementary material 1 [file mgen-8-888-s001.pdf]

SUPPLEMENTARY APPENDIX

One clone to rule them all: Culture-independent genomics of *Chlamydia psittaci* from equine and avian hosts in Australia

Author names

Rhys T. White[1,2,3#], Susan I. Anstey[1#], Vasilli Kasimov[1], Cheryl Jenkins[4], Joanne Devlin[5], Charles El-Hage[5], Yvonne Pannekoek[6], Alistair R. Legione[5], Martina Jelocnik[1*]

Affiliation

[1] University of the Sunshine Coast, Centre for Bioinnovation, Sippy Downs, Sunshine Coast, Queensland 4557, Australia

[2] The University of Queensland, School of Chemistry and Molecular Biosciences, Australian Infectious Disease Research Centre, Brisbane, Queensland 4072, Australia

[3] The University of Queensland, Australian Centre for Ecogenomics, Brisbane, Queensland 4072, Australia

[4] NSW Department of Primary Industries, Elizabeth Macarthur Agricultural Institute, Menangle, New South Wales 2568, Australia

[5] The University of Melbourne, Melbourne Veterinary School, Asia Pacific Centre for Animal Health, Parkville, Victoria 3010, Australia

[6] University of Amsterdam, Amsterdam UMC, Department of Medical Microbiology and Infection Prevention, Amsterdam 1105, The Netherlands

#: These authors have contributed equally.

Corresponding author

*Corresponding author: Martina Jelocnik, University of the Sunshine Coast, Centre for Bioinnovation, Sippy Downs, Queensland 4557, Australia;
Telephone: +61-7-5456-3585; Email: mjelocni@usc.edu.au

<u>This file includes:</u>

Supplementary Figure S1. Genomic comparisons between *Chlamydia psittaci* strain 8882_placenta and strain 9945_foetus and their respective draft assemblies.

Supplementary Figure S2. Evolutionary reconstruction of *Chlamydia psittaci*.

Supplementary Figure S3. Comparison between the phylogeny of the core-genome SNPs and MLST.

Supplementary Figure S4. Phylogenetic relationships of *Chlamydia psittaci* sequence type (ST)24.

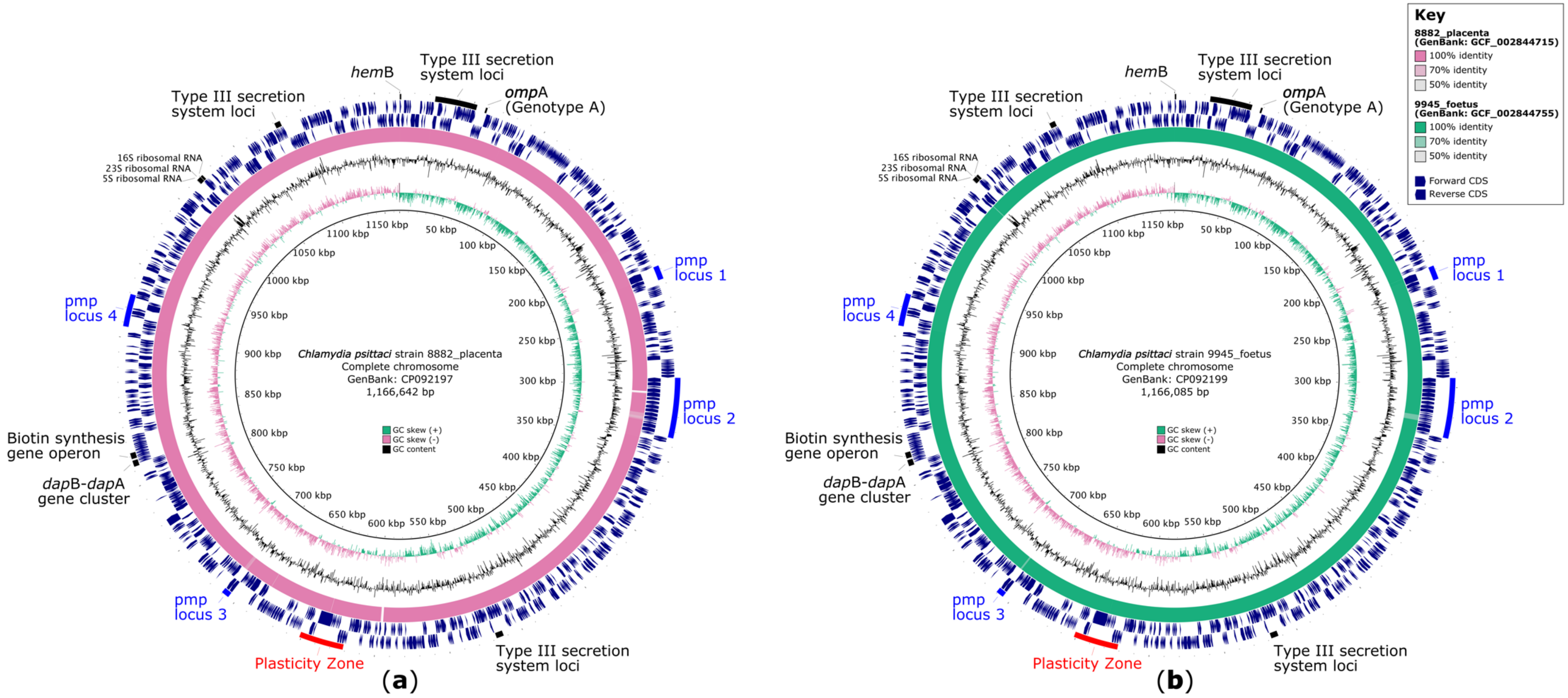

Supplementary Figure S1. Genomic comparisons between *Chlamydia psittaci* strain 8882_placenta and strain 9945_foetus and their respective draft assemblies. (**a**) Circular representation of the chromosome of *C. psittaci* strain 8882_placenta (GenBank: CP092197). (**b**) Circular representation of the chromosome of *C. psittaci* strain 9945_foetus (GenBank: CP092199). The innermost rings represent the chromosome coordinates, GC skew, and GC content. Ring 4 represents nucleotide identity between sequences according to BLASTn (50 to 100%) between the complete chromosome and respective draft assembly. The coding sequence (CDS) for the reverse (ring 5) and forward (ring 6) strand is plotted in navy. Ring 7 highlights regions of interest. Circular genome plots were created using BRIG v0.95 (1).

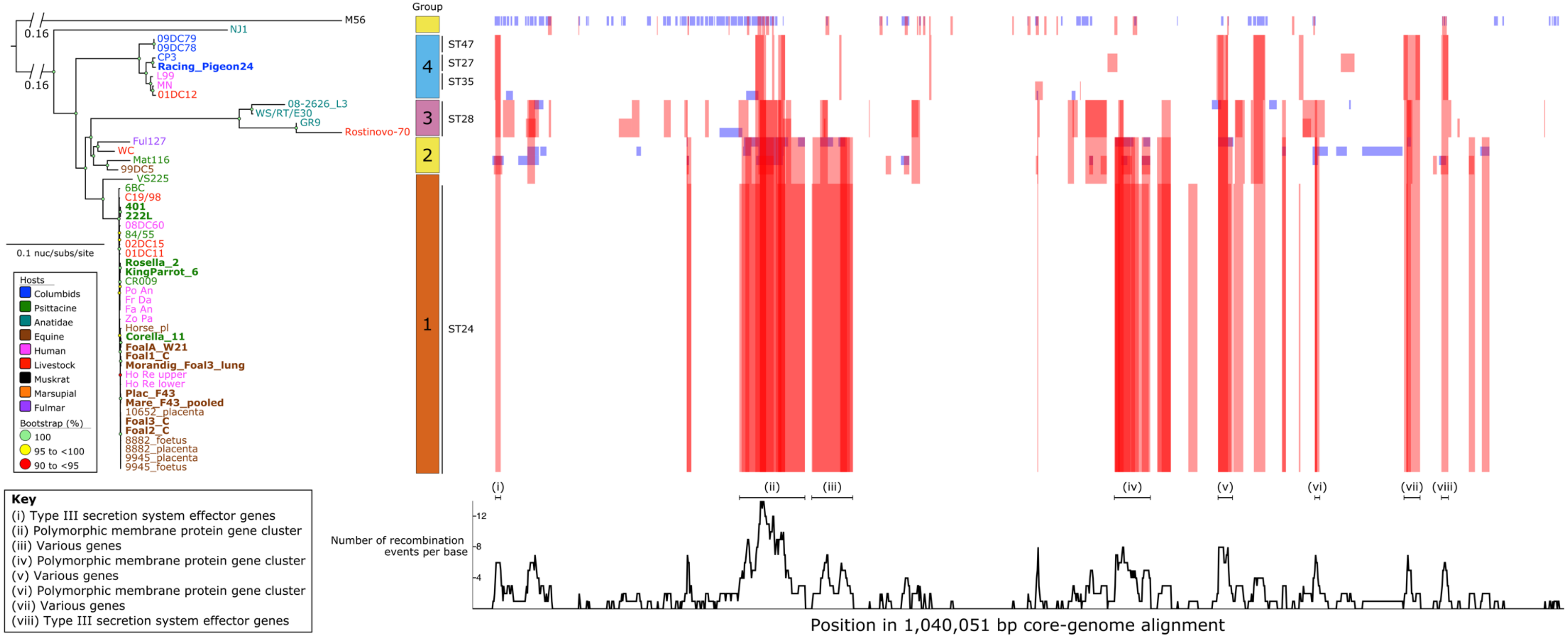

Supplementary Figure S2. Evolutionary reconstruction of *Chlamydia psittaci*. Before recombination filtering, the maximum likelihood phylogeny was inferred from 34,906 core-genome single-nucleotide polymorphisms (SNPs) from 49 genomes. SNPs were derived from a core-genome alignment of 1,040,051 bp and are called against the reference chromosome Horse_pl (GenBank: CP025423). The core-genome phylogeny (left) is plotted against a presence/absence matrix of regions of recombination predicted by Gubbins (right). Blue blocks represent recombination unique to that isolate, whereas red blocks represent ancestral recombination shared by multiple strains. The graphs below the matrix represent the number of recombination events per base. Phylogeny is midpoint rooted. Branch lengths represent the nucleotide substitutions per site, as indicated by the scale bar. Bootstrap values (using 1,000 replicates) are shown.

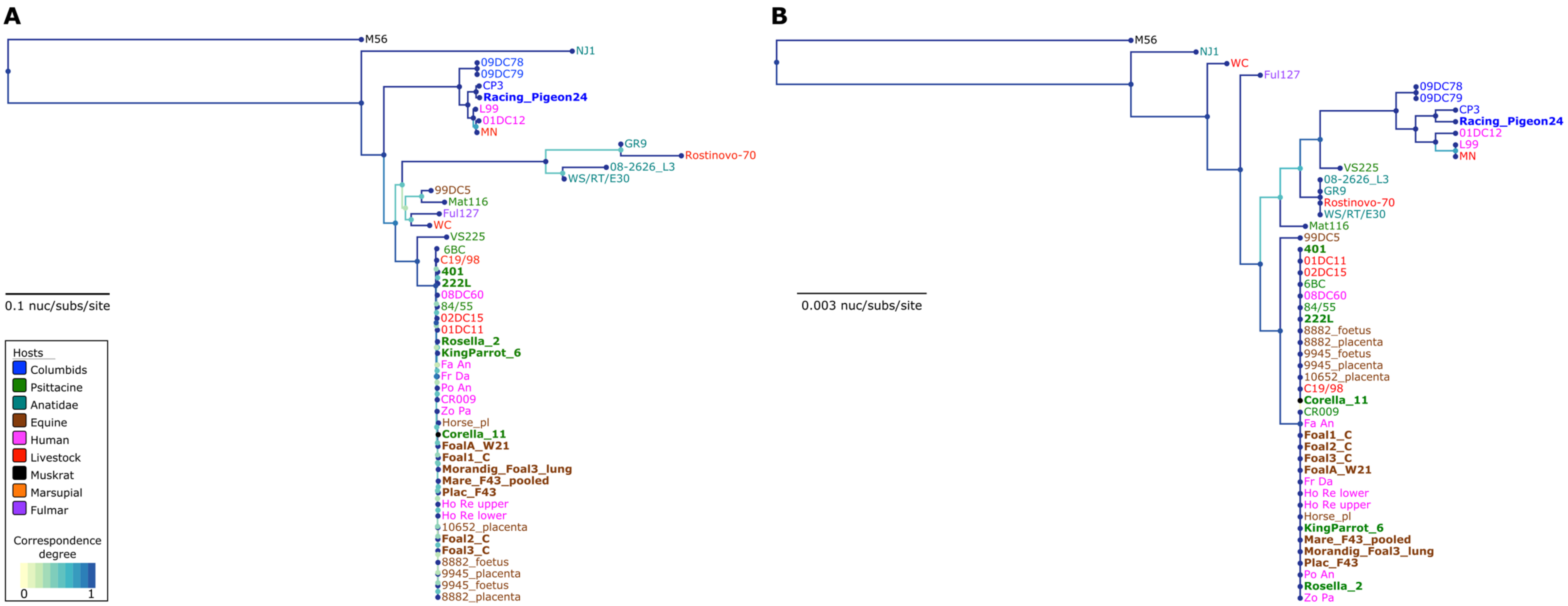

Supplementary Figure S3. Comparison between the phylogeny of the core-genome SNPs and MLST. (A) Comparison of the phylogenetic trees generated from the 30,310 core-genome single-nucleotide polymorphisms (SNPs) and (B) the 3,098 bp alignment representing concatenated multi-locus sequence typing sequences. The colour of the branches represents the comparison metric. A score of 1 denotes that the subtree structure of the node is identical to the subtree structure of its best corresponding node. The figure was performed with the phylo.io tool (2).

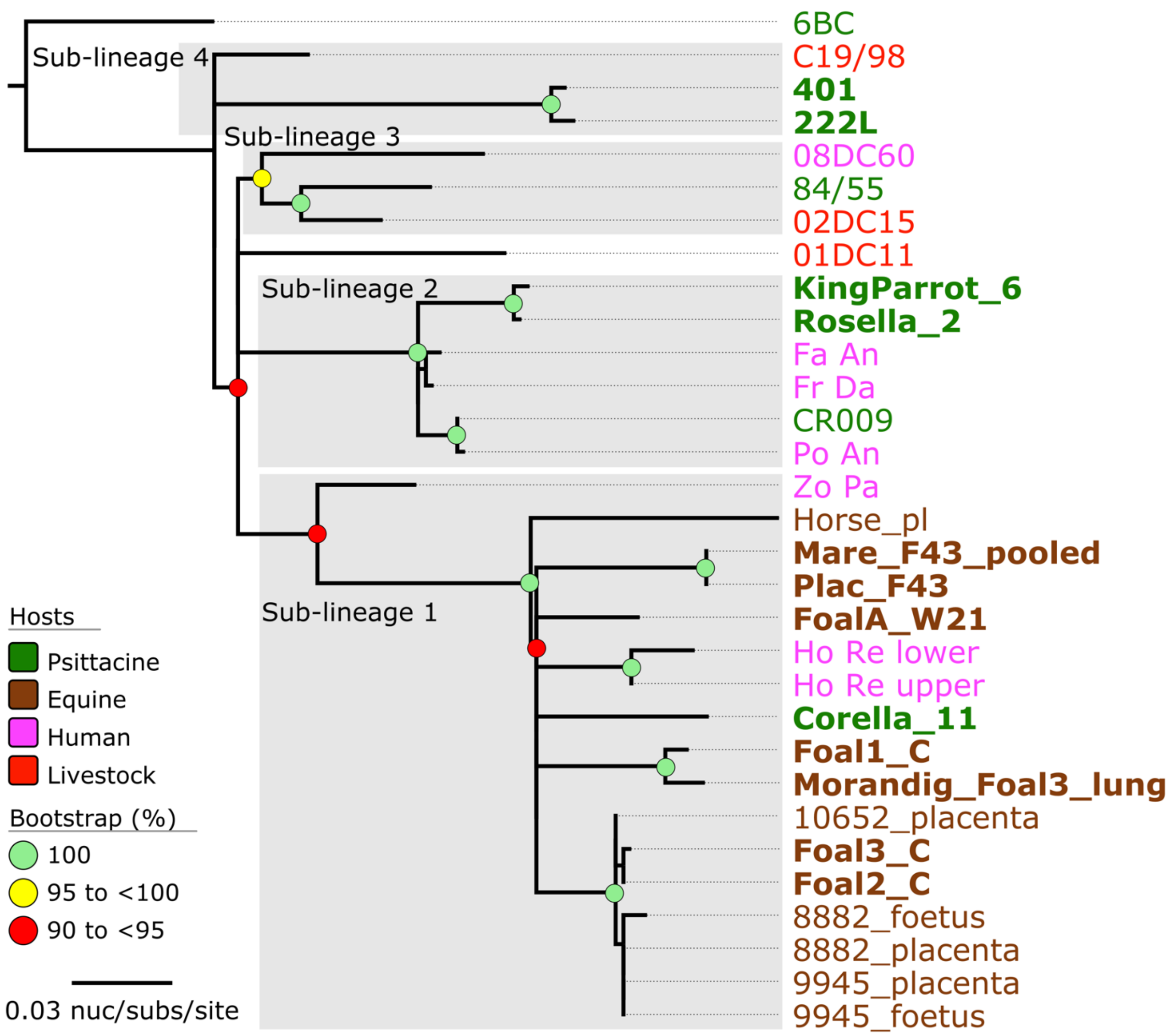

Supplementary Figure S4. Phylogenetic relationships of *Chlamydia psittaci* sequence type (ST)24. Maximum likelihood phylogeny is inferred from 420 core-genome single-nucleotide polymorphisms (SNPs) from 31 genomes. The 420 SNPs were derived from a core-genome alignment of 1,027,103 bp and are called against the reference chromosome Horse_pl (GenBank: CP025423). Phylogeny is rooted according to strain 6BC (GenBank: CP002586). Branch lengths represent the nucleotide substitutions per site, as indicated by the scale bar. Bootstrap values (using 1,000 replicates) are shown. Strains from this study are in bold.

References

1. **Alikhan NF, Petty NK, Ben Zakour NL, Beatson SA**. BLAST Ring Image Generator (BRIG): simple prokaryote genome comparisons. *BMC Genomics* 2011;12:402 doi: 10.1186/1471-2164-12-402

2. **Robinson O, Dylus D, Dessimoz C**. Phylo.io: interactive viewing and comparison of large phylogenetic trees on the web. *Molecular Biology and Evolution* 2016;33:2163-2166 doi: 10.1093/molbev/msw080
